# Neutrophil NETs in reproduction: from infertility to preeclampsia and the possibility of fetal loss

**DOI:** 10.3389/fimmu.2012.00362

**Published:** 2012-11-27

**Authors:** Sinuhe Hahn, Stavros Giaglis, Irene Hoesli, Paul Hasler

**Affiliations:** ^1^Department of Biomedicine, University of Basel/Laboratory for Prenatal Medicine, University Clinics, University Women's HospitalBasel, Switzerland; ^2^Department of Rheumatology, Cantonal Hospital AarauAarau, Switzerland; ^3^Department of Obstetrics, University Clinics, University Women's HospitalBasel, Switzerland

**Keywords:** pregnancy, preeclampsia, infertility, recurrent fetal loss, neutrophil extracellular traps (NETs)

## Abstract

The intention of this review is to provide an overview of the potential role of neutrophil extracellular traps (NETs) in mammalian reproduction. Neutrophil NETs appear to be involved in various stages of the reproductive cycle, starting with fertility and possibly ending with fetal loss. The first suggestion that NETs may play a role in pregnancy-related disorders was in preeclampsia, where vast numbers were detected in the intervillous space of affected placentae. The induction of NETosis involved an auto-inflammatory component, mediated by the increased release of placental micro-debris in preeclampsia. This report was the first indicating that NETs may be associated with a human pathology not involving infection. Subsequently, NETs have since then been implicated in bovine or equine infertility, in that semen may become entrapped in the female reproductive tract during their passage to the oocyte. In this instance interesting species-specific differences are apparent, in that equine sperm evade entrapment via expression of a DNAse-like molecule, whereas highly motile bovine sperm, once free from seminal plasma (SP) that promotes interaction with neutrophils, appear impervious to NETs entrapment. Although still in the realm of speculation it is plausible that NETs may be involved in recurrent fetal loss mediated by anti-phospholipid antibodies, or perhaps even in fetal abortion triggered by infections with microorganisms such as *L. monocytogenes* or *B. abortus*.

## Introduction

Polymorphonuclear neutrophils (**PMNs**) are the most prevalent leucocyte cell type in the peripheral circulation. They are characterized by a uniquely lobulated nuclear structure and a highly granulated cytoplasm. PMNs play a leading role in combatting infection either by phagocytosis or by the release of antibacterial granules (Nathan, 2006). A more recent attribute of these cells is their ability to extrude their DNA into the extracellular environment, as so-called neutrophil extracellular traps (NETs), which serve to ensnare and kill bacteria (Brinkmann et al., 2004; Brinkmann and Zychlinsky, 2007). This unique form of cell death termed NETosis has been shown to be highly reliant on the action of nicotinamide adenine dinucleotide phosphate (NADPH) oxidase, the generation of reactive oxygen species (ROS) (Fuchs et al., 2007), and the combined actions of myeloperoxidase (MPO), neutrophil elastase (NE) (Papayannopoulos et al., 2010), and histone deamination by human peptidylarginine deiminase 4 (PAD4) (Neeli et al., 2008; Wang et al., 2009). It is this facet which forms the basis of this review.

**PMNs** are involved in several stages in the reproductive cycle. In many mammalian species, PMNs have been implicated in tissue remodeling during the oestrous cycle when the endometrium adapts to be receptive for oocyte implantation (Strzemienski, 1989; Wood et al., 2007).

In murine systems, the influx of PMNs into the vaginal vault appears to play a crucial role in the continued progression of the oestrous cycle, as their depletion via application of anti-Gr-1 monoclonal antibodies leads to a blocking of the cycle in diestrus (Sasaki et al., 2009). This blockage was reversible, and could be reverted by the re-introduction of circulatory PMNs into affected mice. This neutropenic abrogation of the oestrous cycle was accompanied by reduction in the levels of the sex hormones oestradiol and progesterone, which are necessary for its maintenance, implying some form of feedback mediated by normal endometrial presence of infiltrating PMNs. The feedback loop was facilitated by opioid peptides released by activated PMNs, which promote steroidogenesis by granulosa cells (Sasaki et al., 2011).

During the human menstrual cycle (MC), large numbers of PMNs have been noted in areas of tissue degradation (day 28) prior to onset of menstruation (Salamonsen and Lathbury, 2000). It appears that endometrial PMNs may have different phenotypes, as not all stained positive for metalloproteinase-9 (MMP-9) (Salamonsen et al., 2000). Endometrial PMNs have also been determined to express ELAFIN, a serine protease inhibitor and member of the whey acidic protein (WAP) family (King et al., 2003a,b). Elafin is a potent inhibitor of NE and proteinase 3, and has been suggested to protect tissue from degradation by these enzymes (King et al., 2003a,b). Since Elafin has been demonstrated to possess anti-microbial activity, its presence could thereby contribute to innate immune defence in the female reproductive tract (FRT) (King et al., 2003a,b).

Hormonal changes occurring during the human MC have also been shown to alter the physiology of circulating PMNs. During the luteal phase of the MC, in which progesterone peaks, a notable increase in circulatory PMNs numbers was noted (Smith et al., 2007). Circulatory PMNs numbers remained high until the onset of menstruation at day 28, at which time they were still higher than those of comparable male donors. During the luteal phase circulatory PMNs exhibited reduced levels of MMP-9 and TNF-α expression (Smith et al., 2007). During periods of oestrogen surges (week 2 and 3 of the MC), circulatory PMNs in women expressed lower levels of CD89 (**FcaR for IgA**), CD11b, and CD18, which returned to normal by week 4. In general, circulatory PMNs in women expressed higher levels of CD89 than those from men (Smith et al., 2007).

The reduction in expression of the integrins CD11b and CD18 could explain the diminution of neutrophil adhesion by oestrogen, and partially explain the anti-inflammatory action of this sex hormone. The generally elevated expression of CD89 on peripheral PMNs by women indicates that they are more responsive to activation via an IgA-based mechanism. Women mostly exhibit a more robust humoral response than men.

In animal systems it is unclear if the oestrus cycle affects circulatory and FRT-associated PMN pro-inflammatory activity. In hormone primed mares no change in the bactericidal activity of circulatory PMNs was noted (Strzemienski et al., 1987).

## Interaction between neutrophils and semen in the female reproductive tract

During coitus billions of sperm are deposited into the FRT. During the sexual act, microorganisms originating from either the penis or the vagina are transported with the ejaculate either into the vagina or directly into the uterus. In order to maintain an environment favorable to implantation, these potential pathogens need to be successfully eliminated. A further task is the removal of the vast majority of sperm, as these can be antigenic to the recipient female, and immunization against sperm can result in infertility. A number of observations have shown that neutrophils are recruited to the FRT following insemination in a manner akin to inflammation, and that they play a major role in the removal of excess sperm, largely via phagocytosis (Strzemienski, 1989; Alghamdi and Foster, 2005; Alghamdi et al., 2009; Katila, 2012).

Even though human reproductive tract leucocytosis has been implicated in human infertility, interactions between neutrophils and sperm have been best studied in large domesticated animals such as cows and horses, due to the frequent use of artificial insemination (AI) for optimal breeding (Katila, 2012). In these instances it has been observed that the repeated deposition of spermatozoa in the presence of neutrophils can lead to diminished fertility (Alghamdi and Foster, 2005; Alghamdi et al., 2009).

In early studies it was noted that bovine seminal plasma (SP) reduced the ability of isolated PMNs to phagocytize bull spermatozoa (Strzemienski, 1989). In further exploratory studies examining the action of SP it was evident that equine SP contained factors that reduced neutrophil binding to spermatozoa *in vitro* (Alghamdi et al., 2004), thereby perhaps permitting a greater number of healthy mobile spermatozoa to reach the oviduct.

In these studies aggregates were noted between large numbers of PMNs and spermatozoa, which could be antagonized by SP. The issue of these PMN-spermatozoa aggregates was subsequently addressed in more detail once it emerged that PMNs were capable of producing extracellular traps (Brinkmann et al., 2004).

Since bovine SP was found to contain a fertility-promoting factor with homology to DNAse I, the question was raised whether such a factor would permit spermatozoa to evade the presence of any PMN NETs in the FRT. In one of the first publications recording the presence of NETs in another system than infection, Alghamdi and colleagues observed that the incubation of isolated peripheral PMNs with equine spermatozoa lead to the vigorous generation of NETs, with kinetics close to those mediated by *E. coli* (Alghamdi and Foster, 2005). They furthermore observed that the protein fraction of equine SP did indeed contain a molecule with DNAse activity, as it was capable of digesting plasmid DNA, in a manner very similar to that performed by DNAse I (Alghamdi and Foster, 2005).

**Figure 1:**
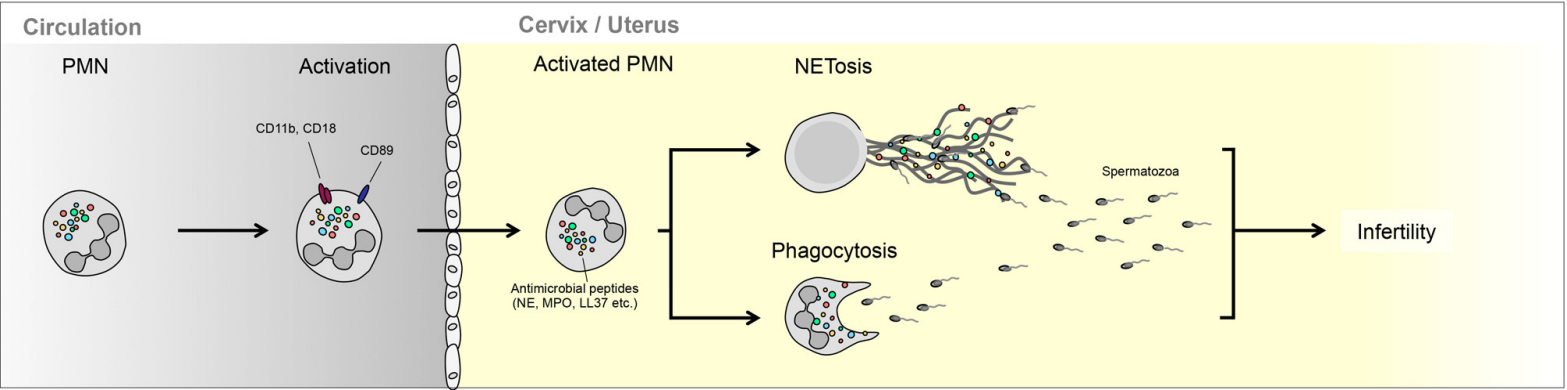
**Interaction between neutrophils and semen in the female reproductive tract.** PMN can either phagocytize less motile spermatozoa or trap these in NETs. The ability of PMNs to interact with spermatozoa is regulated largely by seminal plasma, which can promote NETting (bovine) or enhance escape (equine).

The addition of this equine SP protein fraction to spermatozoa-PMN mixtures led to the digestion of PMN NETs, an aspect that could be partially mimicked by the addition of extraneous DNAse I. It was, however, clear that equine SP contains other factors that modulate PMNs response to spermatozoa, as it reduced the number of NETs generated by accessory PMNs in such cultures (Alghamdi and Foster, 2005) (Figure 1).

Of great interest is that equine SP protein fraction did not prevent NETs induction by *E. coli*, nor did it prevent their killing by activated PMNs. Of further note is that this equine DNAse-like protein was virtually absent in the SP of a stallion with a record of poor fertility. This strongly suggests that this factor assists in mediating optimal interaction between spermatozoa and PMN in the equine FRT, without compromising PMN bacteriocidal activity (Alghamdi and Foster, 2005).

A major difference between bovine and equine insemination is that in the former the spermatozoa are deposited in the vagina, whilst in the latter they are ejaculated directly into the uterus (Alghamdi et al., 2009; Katila, 2012). In bovines the spermatozoa need to migrate through the cervix into the uterus, in the process leaving most of the seminal fluid behind. Therefore, the interaction between SP proteins modulating PMN activity will be diminished in this case. Alternatively, in equines, SP proteins could have a different function, because spermatozoa are discharged adjacent to the site of fertilization.

This indeed turned out to be the case in that bovine SP mediates a different modality on PMN activity than equine SP, by promoting the binding of bovine spermatozoa to PMNs (Alghamdi et al., 2009). On the other hand, in contrast to the situation in horses, bovine spermatozoa cleared completely of SP showed low binding affinity to PMN, for a period as long as 3 h. This implies that less mobile bovine spermatozoa will be ensnared by NETs directly in the bovine vagina, due to the action of SP, while virile highly motile spermatozoa will enter the uterus cleared of SP and thereby evade interaction with any PMNs present in this part of the FRT (Alghamdi et al., 2009).

Apart from demonstrating remarkable species-specific differences, these results have major implications in how AI is carried out, and under which conditions SP protein fractions may have beneficial character or not (Alghamdi et al., 2009; Katila, 2012).

## Occurrence of NETs in preeclampsia

Although the vast majority of human pregnancies progress to term without any complications, many can be affected by disorders such as fetal growth restriction, preeclampsia, or preterm labor, which can result in maternal or fetal mortality/morbidity (Wilkinson, 2011). To a large extent these different pathologies may hold their origin in placental abnormalities (Brosens et al., 2011).

Human placentation is unique by the depth of invasion of the maternal endometrium by fetal tissues, but also by the extent in which maternal blood vessels are modified by fetal trophoblast (Huppertz, 2008; Pijnenborg et al., 2011). In this manner, toward the end of the first trimester, extravillous trophoblast cells enter the maternal endometrium and replace the endothelial cells of the maternal spiral arteries (Redman, 1997). This leads to a significant enlargement and relaxation of these vessels, thereby ensuring an optimal blood flow though the intervillous space.

Failure of such a modification is associated with either severe intra-uterine fetal growth restriction (IUGR) and/or early onset preeclampsia (Redman and Sargent, 2005; Huppertz, 2008; Brosens et al., 2011).

Preeclampsia is a disorder unique to human pregnancy, characterized by proteinuria and sudden elevation in blood pressure in previously normotensive pregnant women (Roberts and Redman, 1993). Currently the only effective therapy is delivery of the baby, which frequently results in extreme prematurity, as the onset of this disorder can occur as early as 20 weeks of gestation. If left untreated, preeclampsia can develop into eclampsia, a potentially lethal condition, characterized by epilepsy-like convulsions (Roberts and Redman, 1993).

Even though the placenta plays a central role in the underlying aetiology (Huppertz, 2008), preeclampsia is associated with systemic damage of the maternal endothelium and an anomalous maternal inflammatory response (Redman and Sargent, 2010). This inflammatory response is mediated in part by the release of placental micro-debris by the placental syncytiotrophoblast, the deportation of which is elevated in cases with preeclampsia (Gupta et al., 2005b; Hahn et al., 2005; Germain et al., 2007; Messerli et al., 2010).

With regard to PMNs, previous studies have shown that normal human pregnancy is associated with a pro-inflammatory phenotype of these cells, a feature which is significantly more pronounced in preeclampsia (Sacks et al., 1998). This was reflected by the elevated expression of cell surfaces markers such as CD11b, but also intracellular activation markers, such as iROS (Sacks et al., 1998). Additional evidence of peripheral PMN activation under these conditions is provided by the presence of elevated levels of NE in the plasma of patients with preeclampsia (Halim et al., 1996; Gupta et al., 2006b). This activation appears to be stimulated by the presence of placental micro-debris in the maternal circulation (Aly et al., 2004).

Prompted by our observations that preeclampsia was associated with significantly elevated fetal and maternal cell-free DNA in the maternal circulation (Zhong et al., 2001b, 2005a,b), we were very intrigued by the report that PMNs can release their nuclear DNA into the extracellular environment (Brinkmann et al., 2004). Consequently, we set out to determine whether a possible connection existed between these two phenomena.

For this purpose we used *in vitro* co-cultures using peripheral PMNs isolated from healthy controls and highly purified placental micro-debris (Gupta et al., 2005a). In our experiments we observed that placental micro-debris led to the activation of PMN as assessed by the elevated expression of CD11b (Gupta et al., 2005a). This activation by placental micro-debris was accompanied by the generation of NETs, in a time and dose dependent manner (Gupta et al., 2005a), with similar kinetics to what had been previously observed using bacterial agents (Brinkmann et al., 2004).

We also observed that NETs could be induced by other placentally derived factors, such as the cytokine IL-8. It is therefore possible that placentally derived micro-debris and inflammatory cytokines (IL-8) may act in concert in the activation of PMNs and induction of NETs in pregnancy (Gupta et al., 2005a).

To assess whether these *in vitro* observations had any physiological relevance we examined placentae from normal healthy term deliveries or those affected by severe preeclampsia. PMN NETs could be detected in the intervillous space of normal placentae. This is to be expected as the normal placenta does deport micro-debris, which could lead to PMNs activation and ensuing NETosis, as part of the pro-inflammatory condition observed in normal pregnancy. The number of NETs in preeclamptic placentae was, however, dramatically elevated and appeared to fill the entire intervillous space in certain instances.

As preeclampsia is characterized by hypoxia-reperfusion damage (Burton and Jauniaux, 2004), the presence of large numbers of NETs directly in the intervillous space, the site of oxygen exchange between mother and fetus, may contribute to this.

Since NETs have recently been shown to promote thrombi in deep vein thrombosis (DVT) and in tumor model systems (Brill et al., 2012; Demers et al., 2012; Fuchs et al., 2012), it is possible that the occlusion of blood flow through the intervillous space by NETs could be exacerbated by such an additional event. The likelihood of such an event is high, as excessive fibrin deposition and infarction are frequently observed in preeclamptic placentae (Kitzmiller and Benirschke, 1973).

PMN NETs may also contribute to the widespread systemic damage to the maternal endothelium observed in preeclampsia (Powe et al., 2011), since endothelial cells are susceptible to cell death induced by NETting PMNs (Gupta et al., 2010).

**Figure 2:**
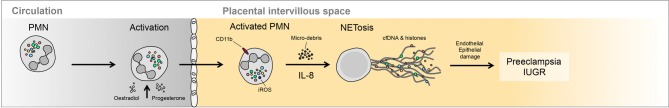
**Occurrence of NETs in preeclampsia.** Large numbers of NETs have been detected directly in the intervillous space of preeclamptic placentae. These NETs appear to be triggered by the elevated release of highly inflammatory placental micro-debris.

Additional support suggesting that placentally derived factors contribute to PMN activation in preeclampsia is provided by the study of circulatory PMNs isolated from maternal blood isolated from the antecubital and uterine veins (Mellembakken et al., 2002). In cases with preeclampsia, it was observed that PMNs passing through the uteroplacental circulation appeared to have a more highly activated phenotype than those present in the peripheral circulation. No similar alteration was observed in normal pregnancies. These data therefore suggest that an inflammatory process occurs in the decidua and placental tissues during the development of preeclampsia (Mellembakken et al., 2002) (Figure 2).

It is currently unclear whether NETs represent an initiating lesion in preeclampsia, or are the result of another underlying placental deficiency. By using the data on maternal and fetal cell-free DNA levels in preeclampsia, it may be possible to tentatively infer regarding the time-point of NETs induction (Gupta et al., 2006a). In this context, elevations in cell-free fetal DNA have been suggested to be the result of a placental lesion, whilst elevations in maternal cell-free DNA could result from the generation of NETs (Gupta et al., 2006a). A number of studies have indicated that disturbances in cell-free fetal DNA levels occur early in gestation, prior to the onset of preeclampsia symptoms (Leung et al., 2001; Zhong et al., 2001a). In contrast, cell-free maternal DNA is only elevated once the symptoms become manifest (Zhong et al., 2001a). These data imply that two phases are present in preeclampsia; a pre-clinical phase involving a placental lesion, and a second clinical phase with symptoms involving a systemic inflammatory response by the mother (Roberts and Hubel, 2009). It would, hence, appear that NETs are associated with the manifest disorder, and not a preclinical initiating lesion (Gupta et al., 2006a).

NETs also do not appear to distinguish between early and late onset preeclampsia, as both seem to be associated with increased PMNs activity as assessed by increased levels of NE in maternal plasma in both forms, when compared to healthy pregnancies (Gupta et al., 2006b).

Currently it is still not clear whether PMN NETs are involved in other pregnancy-related disorders such as IUGR, recurrent fetal loss or preterm labor (Hahn et al., 2006). It is also evident that the underlying aetiology of multi-factorial syndromes, such as preeclampsia, will not involve a single lesion, but rather involve a number of different triggering events (Redman and Sargent, 2005; Brosens et al., 2011). These may include the imbalance of placentally produced angiogenic factors (Powe et al., 2011; Rana et al., 2012), or the dysregulation of inflammatory cytokine networks (Rusterholz et al., 2007; Granne et al., 2011).

## A possible role of NETs in spontaneous fetal loss

Although there is no direct evidence that NETs may be implicated in fetal loss, induced either via the presence of autoantibodies or infectious agents, there is accruing evidence that PMNs activation may play a crucial part in these events (Weiler, 2008; Lynch and Salmon, 2010; Girardi, 2011). Recurrent fetal loss is frequently associated with the presence of maternal antiphospholipid antibodies (aPL) (Weiler, 2008; Lynch and Salmon, 2010; Girardi, 2011). aPL via the interaction with beta-2-glycoprotein-I (β2GPI), binds to phosphotidylserine moieties on the trophoblast, thereby providing a key step for the activation of a series of coagulation factors, hence, the layman's term “sticky blood syndrome.”

The traditional view is that the presence of the aPL leads to activation of the clotting cascade, thereby leading to placental infarction and ensuing fetal demise (Weiler, 2008; Lynch and Salmon, 2010; Girardi, 2011). Support for this notion is provided by the clinical use of low molecular weight heparin, which has been shown to be effective in preventing fetal loss in most cases (Hahn et al., 2006).

Recent evidence indicating that aPL is associated with an inflammatory activation of PMNs via the complement system is leading to a change of the traditional dogma (Salmon and Girardi, 2008).

Evidence for such an interaction was largely provided by murine model systems. It was determined that when aPL were infused into pregnant mice, fetal demise was not associated with a deposition of fibrin or increased presence of thrombi, but rather that it involved the activation of the complement system, in particular components C3 and C5, and the repressive activity of Crry (Holers et al., 2002; Girardi et al., 2003). In addition, the innate arm of the immune system was implicated, as the decidua of treated mice exhibited considerable PMNs infiltration and elevated tissue factor (TF) expression (Redecha et al., 2007).

These findings were largely achieved via the dissection of the underlying molecular pathways using a series of knock-out murine models or pharmacologic inhibitors. In these examinations it was determined that elevated TF expression by PMNs appeared to be a crucial component in triggering fetal demise, which was the result of a complex chain of events (Redecha et al., 2007). The first of these was binding of aPL to the trophoblast, thereby catalyzing zymogenic cleavage of C5. This permits the active component C5a to bind to the C5aR on PMNs. This receptor-ligand interaction triggers increased cell surface expression of TF, itself a receptor for coagulation factors FVIIa and FXa. Such receptor TF/FVIIa-FXa complexes lead to activation of the G-protein coupled protease-activated receptor 2 (PAR-2), which in PMNs initiate ROS production and the release of pro-inflammatory cytokines (Redecha et al., 2007; Weiler, 2008; Girardi, 2011).

PMNs appear to be the final effector in this complement-triggered cascade, as their depletion via the use of appropriate antibodies effectively blocks aPL induced fetal loss (Pierangeli et al., 2005). For this reason it was suggested aPL induced fetal loss was the result of uncontrolled ROS production by complement activated PMNs in the feto-maternal interface, leading to oxidative damage of underlying placental tissue (Salmon and Girardi, 2008).

As the production of ROS is a vital component of the pathway triggering NETosis and the release of DNA into the extracellular environment (Fuchs et al., 2007), it is enticing to speculate that NETs may occur in aPL induced fetal loss. Furthermore, as the presence of NETs can be cytotoxic to closely adjacent cells (Gupta et al., 2010), it is possible that the occurrence of such entities can contribute to trophoblast injury apparent in this disorder.

An interesting observation is that PMNs from preterm infants or neonates do not appear capable of undergoing NETosis, or at least showed a very delayed response, when treated with potent stimuli such as platelet activating factor (PAF) and lipopolysaccharide (LPS), unlike normal adult PMNs (Marcos et al., 2009; Yost et al., 2009). This facet did not appear to involve a defect in signaling pathways, as neonatal PMNs expressed the required receptors (PAF-R and TLR4) and displayed normal responses, such as calcium mobilization, production of IL-8 in response to these stimuli. This deficit could also not be overcome by supplementation of intracellular ROS pools by treatment with glucose/glucose oxidase. It is not clear from this particular study how representative the relatively inert nature of neonatal PMN in cases with preterm delivery is, since previous reports have suggested that intrauterine activation of PMNs occurs under these conditions, leading to pulmonary haemorrhage in affected infants (Mehta and Petrova, 2006).

Although it may seem that the coagulation cascade is not directly involved in this particular model system, since fetal loss could not be hindered by anti-clotting agents such as hirudin, which prevent thrombus formation but not complement activation, it may nevertheless play an accessory role. This would especially be the case if NETs were implicated in aPL induced fetal loss, as these could provide the necessary stimulus and scaffold for clot formation (Fuchs et al., 2012). This supposition is based on recent data indicating that NETs stimulate the extrinsic and intrinsic coagulation pathways, by promoting platelet and RBC adhesion and by concentrating effector proteins and coagulation factors involved in hemostasis (Fuchs et al., 2010, 2012; Massberg et al., 2010; von Bruhl et al., 2012). In this context NETs have been found to be abundant in experimental DVT in baboons and mice, co-localizing with vWF, an important endothelial clotting factor (Brill et al., 2012). Apart from providing a DNA-based scaffold facilitating the binding of pro-coagulatory factors such as Factor XII, intrinsic PMN derived serine proteases such as NE and cathepsin G can stimulate clotting via the cleavage of coagulation mediators such as tissue factor pathway inhibitor (TFPI), or fibrin (Plow, 1980; Higuchi et al., 1992; Massberg et al., 2010; Semeraro et al., 2011).

Given that preeclampsia, IUGR and even fetal loss are broadly related to dysfunctions at the interface between innate immunity and haemostasis, it would be of cardinal importance to investigate the potential triggers of PMNs activation and NETosis in these pathologies. The trigger could be the interaction of neutrophils with activated cells, such as platelets or endothelium (Gupta et al., 2010; Saffarzadeh et al., 2012), or alternatively, involve hypoxia, inflammatory cytokines, or factors generated early in thrombotic events (Fuchs et al., 2007).

**Figure 3:**
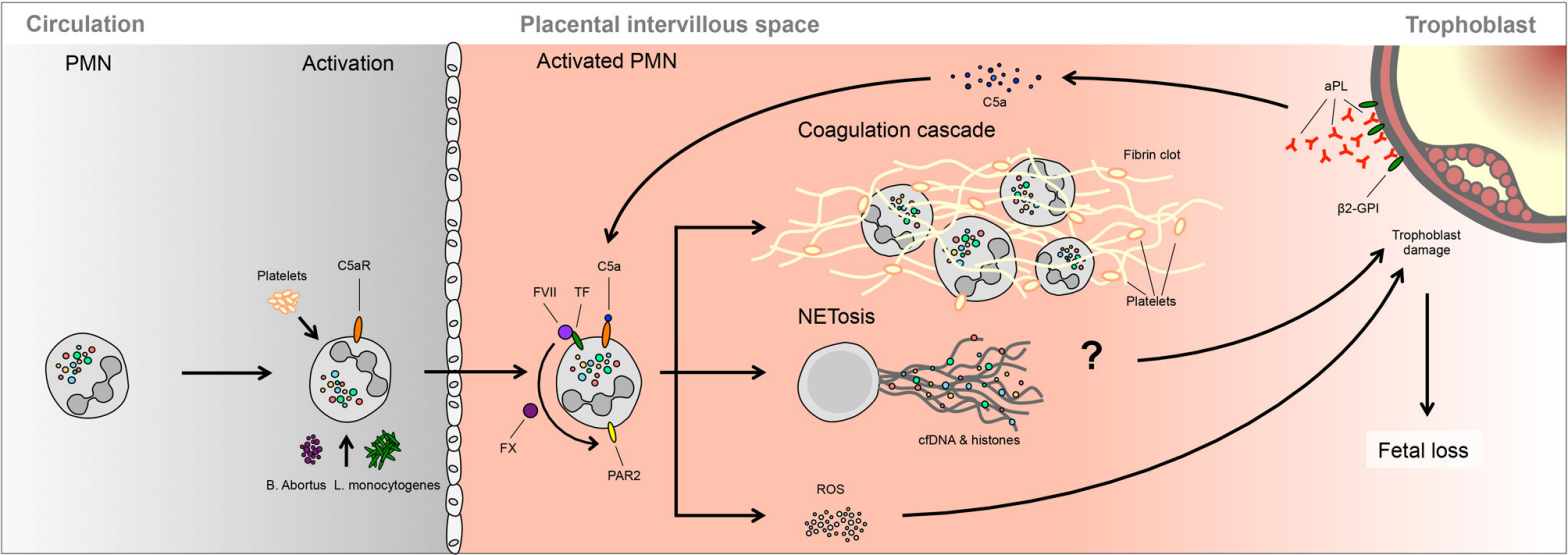
**A possible role of NETs in spontaneous fetal loss.** In murine models, fetal loss triggered by aPL involves activation of the complement cascade, notably C3 and C5, which lead to PMNs activation via TF. Ensuing ROS production is proposed to lead to trophoblast damage, leading to fetal demise. It is not clear whether this process involves NETosis or PMNs activation of the clotting cascade.

The scope of PMN activity associated with fetal loss may, however, be even broader. This is based on recent data suggesting that infections with *Brucella abortis* (Gupta and Bianchi, 1997) or *Listeria monocytogenes* (Knowles et al., 2011) leads to PMNs recruitment and activation, including release of IL-8 and ROS production. As *brucellosis* in cattle or *listeriosis* in humans can be directly associated with spontaneous abortion (Robbins and Bakardjiev, 2012), it is open to speculation whether NETs occur in infected placentae in these conditions, and thereby contribute to the process of fetal loss (Figure 3).

## Conclusions

The activity of PMNs is modulated or altered during various phases of the reproductive cycle (Salamonsen and Lathbury, 2000; Wood et al., 2007; Sasaki et al., 2009). This is most evident in the menstrual or oestrum cycle, in which the activity of circulatory PMNs is altered by the action of sex hormones, but also in the endometrial tissue, where PMNs play a role in tissue removal or modification. PMNs appear to play a significant role in post mating inflammatory response (Katila, 2012), as well as in the trapping and clearance of sperm by NETs (Alghamdi et al., 2004; Alghamdi and Foster, 2005). In these instances, the action of seminal fluid reveals interesting species specific differences, in that in horses it down-modulates PMN activity, thereby promoting sperm escape, whereas in cattle it promotes sperm entrapment by NETs (Alghamdi et al., 2004; Alghamdi and Foster, 2005).

The presence of large numbers of NETs directly in the intervillous space in preeclampsia (Gupta et al., 2005a), a crucial interface between mother and fetus, as well as the induction of NETosis by placentally-derived micro-vesicles or cytokines, suggest a role for these entities in this enigmatic disorder (Gupta et al., 2007).

Although still in the realm of speculation, new evidence implicating PMNs activation in fetal loss induced by auto-antibodies (Salmon and Girardi, 2008) or infectious agents (Robbins and Bakardjiev, 2012), may involve the occurrence of NETs.

### Conflict of interest statement

The authors declare that the research was conducted in the absence of any commercial or financial relationships that could be construed as a potential conflict of interest.

## Abbreviations

FRT: female reproductive tract
PMN: polymorphonuclear neutrophil
MC: menstrual cycle
SP: seminal plasma
AI: artificial insemination
IUGR: intra-uterine fetal growth restriction
NE: neutrophil elastase
aPL: antiphospholipid antibodies
β2GPI: beta-2-glycoprotein I
PAR-2: protease-activated receptor 2
ROS: reactive oxygen species.
